# Subthreshold nitric oxide synthase inhibition improves synergistic effects of subthreshold MMP‐2/MLCK‐mediated cardiomyocyte protection from hypoxic injury

**DOI:** 10.1111/jcmm.12827

**Published:** 2016-03-17

**Authors:** Iwona Bil‐Lula, Han‐Bin Lin, Dariusz Biały, Magdalena Wawrzyńska, Lucas Diebel, Jolanta Sawicka, Mieczyslaw Woźniak, Grzegorz Sawicki

**Affiliations:** ^1^ Department of Clinical ChemistryWroclaw Medical UniversityWroclawPoland; ^2^ Department of PharmacologyCollege of MedicineUniversity of SaskatchewanSaskatoonSKCanada; ^3^ Department and Clinic of CardiologyWroclaw Medical UniversityWroclawPoland; ^4^ Department of Emergency MedicineWroclaw Medical UniversityWroclawPoland

**Keywords:** hypoxia–reoxygenation, cardiomyocytes, synergism, nitric oxide, phosphorylation, contractile proteins, matrix metalloproteinase‐2

## Abstract

Injury of myocardium during ischaemia/reperfusion (I/R) is a complex and multifactorial process involving uncontrolled protein phosphorylation, nitration/nitrosylation by increased production of nitric oxide and accelerated contractile protein degradation by matrix metalloproteinase‐2 (MMP‐2). It has been shown that simultaneous inhibition of MMP‐2 with doxycycline (Doxy) and myosin light chain kinase (MLCK) with ML‐7 at subthreshold concentrations protects the heart from contractile dysfunction triggered by I/R in a synergistic manner. In this study, we showed that additional co‐administration of nitric oxide synthase (NOS) inhibitor (1400W or L‐NAME) in subthreshold concentrations improves this synergistic protection in the model of hypoxia–reoxygenation (H‐R)‐induced contractile dysfunction of cardiomyocytes. Isolated cardiomyocytes were subjected to 3 min. of hypoxia and 20 min. of reoxygenation in the presence or absence of the inhibitor cocktails. Contractility of cardiomyocytes was expressed as myocyte peak shortening. Inhibition of MMP‐2 by Doxy (25–100 μM), MLCK by ML‐7 (0.5–5 μM) and NOS by L‐NAME (25–100 μM) or 1400W (25–100 μM) protected myocyte contractility after H‐R in a concentration‐dependent manner. Inhibition of these activities resulted in full recovery of cardiomyocyte contractility after H‐R at the level of highest single‐drug concentration. The combination of subthreshold concentrations of NOS, MMP‐2 and MLCK inhibitors fully protected cardiomyocyte contractility and MLC1 from degradation by MMP‐2. The observed protection with addition of L‐NAME or 1400W was better than previously reported combination of ML‐7 and Doxy. The results of this study suggest that addition of NOS inhibitor to the mixture of inhibitors is better strategy for protecting cardiomyocyte contractility.

## Introduction

Coronary reperfusion has become the standard procedure for the treatment of patients with myocardial infarction (MI) 1. However, the efficacy of this procedure is limited by the reperfusion‐induced increased expression of inducible or endothelial nitric oxide synthases (iNOS and eNOS), the subsequent production of toxic peroxynitrite (ONOO^−^) 2, 3 and the activation of matrix metalloproteinases (MMPs) 4, 5, 6. Consequently, studies on the pharmacological prevention and/or therapy of MI are still relevant.

It is well known that the mechanism of myocardial injury during ischaemia/reperfusion (I/R) is complex and multifactorial issue, leading to metabolic, morphological and contractile disorders. It is established that during I/R, reactive oxygen species (ROS), such as peroxynitrite (ONOO^−^), activate MMP‐2 6 that degrades contractile proteins 7, 8, 9 and nitrates/nitrosylates myosin light chain 1 (MLC1), which enhances its degradation by MMP‐2 10, 11. ONOO^−^ is formed at an extremely rapid rate from reaction of nitric oxide with superoxide (O_2_^−^), and nitric oxide formation is catalysed by NOS 10. Within the normal heart, nitric oxide is synthesized by eNOS 11 and acts to maintain a variety of physiological functions such as setting coronary vasodilator tone, modulating myocardial contractile function and providing an antioxidant environment 12. However, under pathological conditions, nitric oxide can be cytotoxic 13 and has been associated with the upregulated inflammatory response observed in I/R, septicaemia, ageing and heart failure 14. The increased expression of iNOS under these conditions results in the production of an increased amount of ONOO^−^.

We showed that oxidative stress, during hypoxia–reoxygenation (H‐R), I/R, or asphyxia, induces nitration/nitrosylation 15, 16, 17 and phosphorylation 18 of myocardial contractile proteins, such as MLC1 and MLC2 15, 16, 17, 18. Furthermore, we showed that those modifications led to an increase in degradation of these proteins by MMP‐2 resulting in contractile dysfunction 15, 16, 17, 18. Finally, in these studies, we also showed that preventing these modifications protects hearts from I/R injury 16, 18.

Recently, we demonstrated that in addition to the pathological role of MMP‐2 in I/R, MMP‐2 also regulates MLC levels under physiological conditions 15, 19. We showed that the complete inhibition of intracellular MMP‐2 in aerobically perfused cardiomyocytes increases MLC levels and increases cardiomyocyte contractility above normal 19. Aside from increased phosphorylation and nitration/nitrosylation of MLC and its degradation by MMP‐2 in pathological conditions, other researchers showed that these modifications of MLC are important for its physiological roles 20, 21, 22, 23, 24. Nitration of MLC has been reported in the process of vascular ageing 20, 21, and the phosphorylation of MLC2 controls the sensitivity of myofilaments to calcium in the regulation of cardiac contractility 22, 23, 24.

Given that both MMP‐2 activity and MLC modifications have important physiological roles, full pharmacological blockade would result in a multitude of physiological side‐effects that may be as detrimental as the oxidative stress–induced injury itself. Thus, we propose that instead of using a single drug at its effective concentration, the use of a combination of drugs, each at a concentration below their effective concentration, may afford similar, if not better, protection while limiting adverse physiological side‐effects. These agents will have different modes of action and can synergistically or additively protect the heart from oxidative stress–induced injury.

We already showed that simultaneous inhibition of the activities of MMP‐2 and MLC kinase (MLCK) with subthreshold concentrations of their inhibitors [doxycycline (Doxy) for MMP‐2 and ML‐7 for MLCK] protected I/R hearts from contractile dysfunction 25. In the present study, we hypothesize that adding inhibitors of NOS to the previously established mixtures of MMP‐2 and MLCK inhibitors will provide greater protection of the heart from ischaemic injury than the mixture of MMP‐2 and MLCK inhibitors only.

As there is no universally accepted pharmacological approach that gives satisfactory protection from, or treatment of, reperfusion injury, new directions in pharmacological treatment of patients with cardiac injury are needed. Consequently, it is important to unravel the mechanisms involved in heart injury and to develop new pharmacological strategies for the prevention and treatment of heart diseases.

Here, we hypothesize that the pretreatment of isolated cardiomyocytes with a cocktail comprised subthreshold concentrations of Doxy, ML‐7 and 1400W (a selective inhibitor of iNOS) or L‐NAME (non‐selective inhibitor of NOS) will completely protect cardiomyocytes from H‐R‐induced injury.

## Materials and methods

This investigation conforms to the Guide to the Care and Use of Experimental Animals published by the Canadian Council on Animal Care. All studies involving animals are reported in accordance with the ARRIVE guidelines for reporting experiments involving animals 26, 27.

### Animals maintaining

The hearts were excised from 1‐ to 1.5‐month‐old male Sprague‐Dawley rats weighting 100–130 g, supplied by Charles River Canada. These animals were housed in pathogen‐free facility and individually ventilated cages under a 12 hr/12 hr light/dark cycle (lights on 12 hrs) with controlled room temperature (21–22°C) and humidity (not regulated) and were allowed *ad libitum* access to a diet of standard laboratory chow and water.

### Heart extraction

The hearts were rapidly excised from rats anaesthetized with sodium pentobarbital (40 mg/kg, i.p.). Spontaneously beating hearts rinsed by the immersion in the ice‐cold Myocyte Isolation Buffer (MIB) containing 120 nM NaCl, 5 mM KCl, 2 mM NaAc, 2 mM MgCl_2_, 1 mM Na_2_HPO_4_, 20 mM NaHCO_3_, 5 mM glucose, 9 mM taurine and 10 mM CaCl_2_ at pH 7.4 immediately after removal were suspended on a blunt end needle of Langendorf system with the aorta and maintained at 37°C. Hearts were perfused in a water‐jacketed chamber of the Langendorf mode at a constant flow of 10 ml/min. with MIB buffer containing 10 mM CaCl_2_, pH 7.4, at 37°C and gassed continuously with 5% carbogen for 5 min.

### Myocyte isolation

After 5 min. of heart perfusion with MIB containing 1 mM CaCl_2_, the buffer was replaced with MIB containing 5 μM CaCl_2_ and the hearts were perfused for 5 more minutes as before. The low concentration of CaCl_2_ induced the loss of contractility of cardiomyocytes. After mild swelling of myocardium with HEPES buffer (120 mM NaCl 140, 5 mM KCl, 2 mM MgCl_2_, 5 mM glucose, 9 mM taurine, 5 mM HEPES) containing 40 μM CaCl_2_, 25 mg of collagenase and 2 mg of protease at pH 7.4, the right ventricle was excised from the heart, rinsed with HEPES buffer containing 100 μM CaCl_2_, 150 mg bovine serum albumin (BSA), and then minced into small pieces in the digestion solution (HEPES buffer containing 100 μM CaCl_2_, 150 mg BSA, 15 mg collagenase and 1 mg protease). Minced tissue was repeatedly digested [5–6 times for 20 and 10 min. in water bath (37°C)], and 3rd–5th fraction was used for further experiments.

### Chemical hypoxia

**Figure 1:**
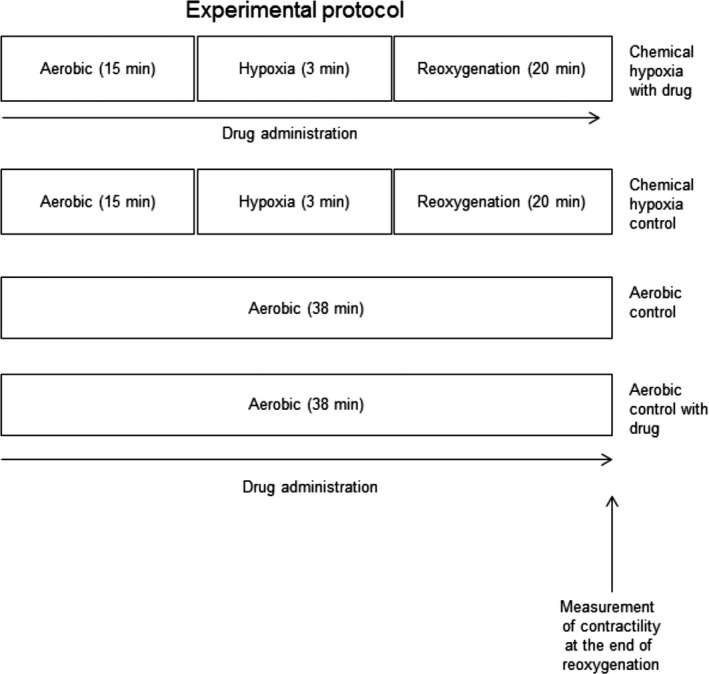
Experimental protocol for chemical hypoxia–reoxygenation (H‐R) and aerobic control with or without drug treatment. Isolated cardiomyocytes were incubated with Doxy (10–100 μM) or ML‐7 (0.5–5 μM) or L‐NAME (25–100 μM) or 1400W (25–100 μM) or with subthreshold doses of Doxy (10 μM) + ML‐7 (0.5 μM) + L‐NAME (25 μM) or 1400W (25 μM) for 15 min. before and 20 min. after chemical ischaemia.

The scheme of the experimental protocols is shown in Figure 1. Briefly, chemical hypoxia (H) was induced after 15 min. of drug treatment (10–100 μM Doxy, 0.5–5 μM ML‐7, 25–100 L‐NAME μM or 25–100 μM 1400W in HEPES buffer containing 100 μM CaCl_2_, 150 mg BSA) by covering the cell pellets with HEPES buffer containing 4 mM 2‐deoxyglucose and 40 mM sodium cyanide (2.5 μM). The optimal duration of ischaemia, 3 min., was established in previous studies 14. Three‐minute ischaemia caused approximately 50% loss in cell contractility, and viability of cells was maintained at the level of 70% or higher 19. After 3 min. of incubation, the buffer containing sodium cyanide was removed by centrifugation (1 min. 1500 × g) and the cells pellet was resuspended in the fresh portion of HEPES buffer containing 100 μM CaCl_2_, 150 mg BSA and appropriate drug. After reoxygenation (R), the cells were centrifuged 1500 × g for 5 min. and the cells pellet, resuspended in HEPES buffer (100 μM CaCl_2_, 150 mg BSA), was used for contractility measurement or rapidly frozen at −80°C for further analysis.

The aerobic control group was kept exposed to atmospheric air for 38 min., and the chemical hypoxia control group cardiomyocytes underwent the same experimental protocol without drug treatment.

### Cardiomyocytes contractility measurement

The contractility of cardiomyocytes was measured at the end of the protocols featured on Figure 1. A 100‐μl aliquot of cell suspension was placed in the rapid change stimulation chamber of the IonOptix Contractility System (IonOptix, Milton, MA, USA). After 3 min. of stabilization, the cardiomyocytes were perfused with oxygenated HEPES buffer containing 2 mM CaCl_2_ (4 ml/min.) at 37°C. Cells were continuously paced with 1 Hz and 5 V (IonOptix MyoPacer, Milton, MA, USA), and the contractility, expressed as a per cent of peak shortening in comparison to the length of the diastolic cell, was measured on an average of 5 cells per sample. At least four samples per one experimental condition were evaluated.

### Preparation of cardiomyocyte extracts

At the end of experimental protocol, cardiomyocytes were homogenized by sonication (twice for 5 sec.) on ice in 50 mM Tris‐HCl (pH 7.4), containing 3.1 mM sucrose, 1 mM DTT, 10 μg/ml leupeptin, 10 μg/ml soybean trypsin inhibitor, 2 μg/ml aprotinin and 0.1% Triton X‐100. Homogenates were centrifuged at 10,000 × g, for 10 min., at 4°C, and the supernatant was stored at −80°C for further biochemical analysis.

The total protein content in cardiomyocyte extracts was analysed by the Bradford based protein assay from Bio‐Rad (Hercules, CA, USA) and standardized with bovine serum albumin.

### Measurement of iNOS/eNOS activity

The activity of NOS in cardiomyocytes was indirectly assessed by means of nitrate and nitrite measurement (the oxidized forms of nitric oxide) in cardiomyocytes subjected to H‐R in comparison to control cells. For this reason, the quantitative Nitric Oxide Assay Kit (Abcam, Cambridge, MA, USA) was used. Briefly, in a two‐step reaction, nitrates were converted into nitrites by nitrate reductase and then nitrites were coupled into colour azo compound with maximum absorbance at 540 nm. Nitric oxide content in cardiomyocytes was expressed as nM/mg of total protein.

### Immunoblot analysis

Myosin light chain 1 content in extracts of cardiomyocytes was determined by Western blot. An aliquot containing 20 μg of total proteins extracted from cardiomyocytes was analysed on 12% SDS‐PAGE. Myosin light chain 1 transferred on PVDF membrane (Bio‐Rad) was detected with mouse monoclonal anti‐MLC1 antibody (Abcam) and Alexa fluor 488 goat antimouse IgG (Invitrogen, Eugene, OR, USA). As an additional control of protein loading, we measured tubulin level. Tubulin was detected with mouse monoclonal antibody (Abcam). VersaDoc 5000 and Quantity One software (Bio‐Rad) were used for fluorescence and band density measurement. MLC1 level is expressed as densitometric units of MLC‐1 level divided by densitometric units of tubulin level.

### Statistical analysis

For contractility measurements, at least six independent experiments (myocyte preparations from different hearts) were run. Each experiment was performed in quadruplicate (myocytes from the same heart). anova with Kruskal–Wallis post hoc analysis or Student's *t*‐tests was used in this study. *P <* 0.05 indicated statistical significance. Data are presented as the mean ± S.E.M.

## Results

To check the potential harmful effect of used drugs on cardiomyocytes contractility, the cardiomyocytes were subjected to the protective concentrations (highest concentrations used in this study) of Doxy, ML‐7, L‐NAME or 1400W in aerobic condition (see Fig. 1 to see experimental protocol). None of the tested agents in this experimental condition had an effect on cardiomyocyte contractility.

### Effects of inhibitors of MMP‐2 and MLCK activities on cardiomyocyte contractility

**Figure 2:**
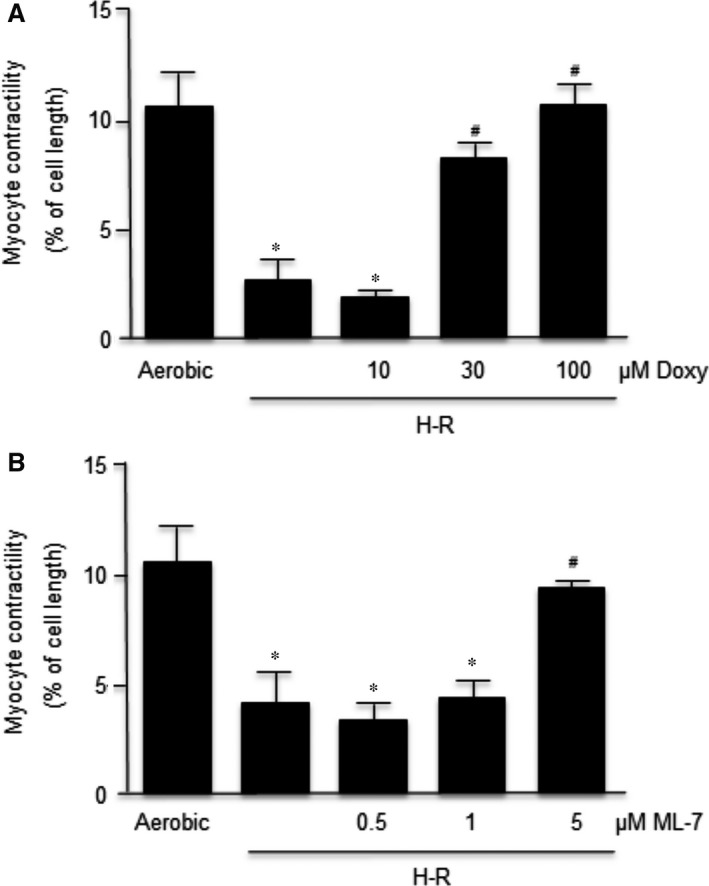
Influence of Doxy (**A**) and ML‐7 (**B**) in a dose‐dependent manner on contractility of cardiomyocytes. The contractility was expressed as peak shortening (%) in comparison to the length of the diastolic cell. *n* = 6/group; *P < 0.05 in comparison to aerobic control; ^#^ *P* < 0.05 in comparison to H‐R control.

The cardiomyocyte contractility, expressed as a peak shortening (% of cell length), was significantly decreased (>70%) in cells subjected to hypoxia followed by re‐oxygenation in comparison to cardiomyocytes subjected to aerobic conditions (Fig. 2). Doxy and ML‐7 inhibitors protected cell contractility in a concentration‐dependent manner (Fig. 2). Doxy at 100 μM concentration resulted in the full protection of contractility, whereas 10 μM represents a subthreshold concentration that did not protect the mechanical function of cardiomyocytes exposed to H‐R (Fig. 2A). ML‐7 at 5 μM concentration showed full protection of contractility, whereas 0.5 μM did not protect H‐R cardiomyocytes and thus is a subthreshold concentration (Fig. 2B).

### Effect of inhibitors of NOS activity on recovery of cardiomyocyte contractility

**Figure 3:**
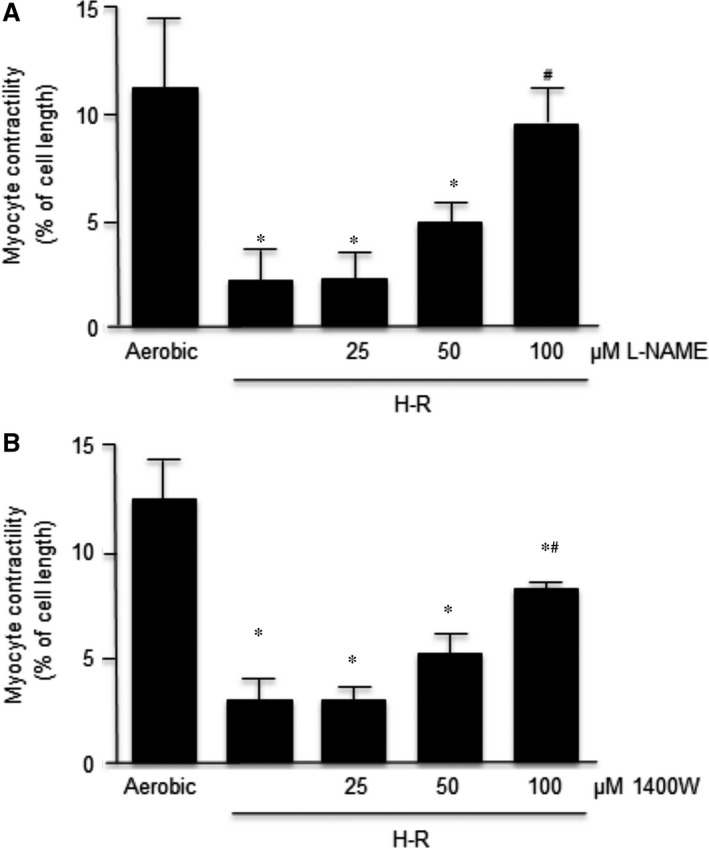
Influence of L‐NAME (**A**) and 1400W (**B**) in a dose‐dependent manner on contractility of cardiomyocytes. The contractility was expressed as peak shortening (%) in comparison to the length of the diastolic cell. *n* = 6/group; *P < 0.05 in comparison to aerobic control; ^#^ *P* < 0.05 in comparison to H‐R control.

The effect of L‐NAME (a non‐selective inhibitor of NOS) and 1400W (a selective inhibitor of iNOS) on contractility of isolated cardiomyocytes is shown in Figure 3. Both inhibitors protected cell contractility in a concentration‐dependent manner. L‐NAME at 100 μM concentration produced full protection of contractility, whereas 25 μM is a subthreshold concentration that did not protect the mechanical function of H‐R cardiomyocytes (Fig. 3A). At a 100 μM concentration, 1400W produced approximately 70% protection of contractility. However, 25 μM of 1400W did not protect against H‐R‐induced reduction in contractility and thus is a subthreshold concentration (Fig. 3B).

### The effect of NOS inhibitors on nitric oxide production

**Figure 4:**
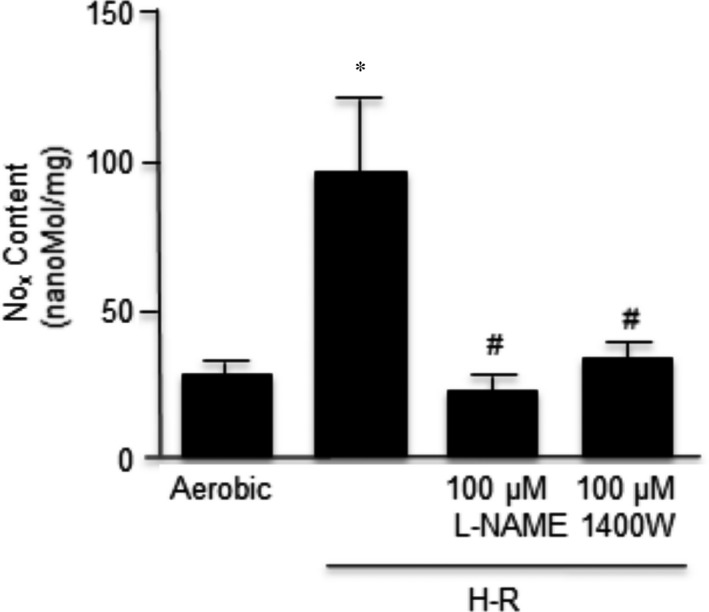
Effect of L‐NAME and 1400W on nitric oxide synthesis in cardiomyocytes. Nitric oxide content was expressed as nM/mg of total protein. *n* = 6/group; *P < 0.05 compared to aerobic controls; ^#^ *P* < 0.05 in comparison to H‐R control.

Both L‐NAME and 1400W reduced nitric oxide synthesis. As shown in Figure 4, the synthesis of nitric oxide in cardiomyocytes subjected to H‐R was three‐fold higher than in cells undergoing aerobic conditions. The non‐selective inhibitor of NOS (L‐NAME) and the selective iNOS inhibitor (1400W) led to a significant reduction of nitric oxide in cardiomyocytes subjected to H‐R, and those values were similar to the cells not subjected to H‐R.

### Effect of co‐administration of subthreshold doses of inhibitors of MMP‐2, MLCK and NOS on myocyte contractile function

The primary objective of this study was to determine if the use of a combination of subthreshold concentrations of drugs affecting three different pathways of cardiac injury would protect the contractile function of myocytes subjected to H‐R. For this study, isolated cardiomyocytes were pretreated with the mixture of subthreshold concentrations of two drugs: Doxy (10 μM) and ML‐7 (0.5 μM) or three drugs: Doxy (10 μM), ML‐7 (0.5 μM) and L‐NAME (25 μM) or 1400W (25 μM) for a total of 38 min. during the experimental protocol.

**Figure 5:**
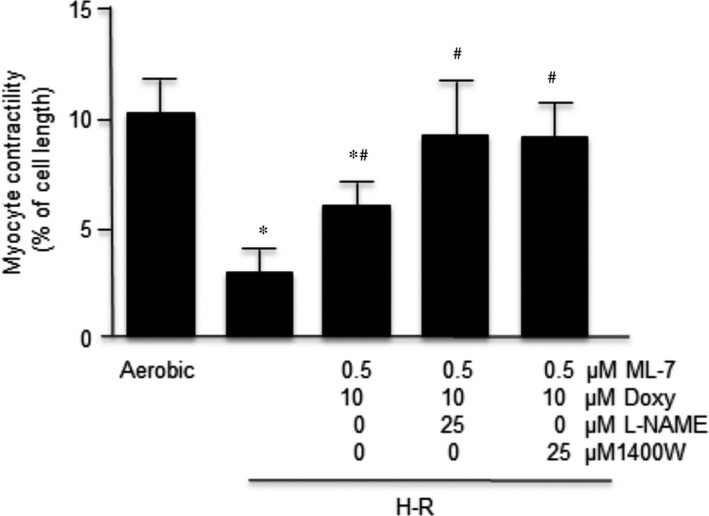
Synergistic effect of combined subthreshold doses of ML‐7, Doxy, L‐NAME/1400W on contractility of cardiomyocytes. The contractility was expressed as peak shortening (%) in comparison to the length of the diastolic cell. *n* = 6/group; *P < 0.05 in comparison to aerobic control; ^#^ *P* < 0.05 in comparison to H‐R control.

As shown in Figure 5, pretreatment with subthreshold Doxy plus ML‐7 combination partially protected cell contractility. Pretreatment with Doxy plus ML‐7 plus either NOS inhibitor fully protected the contractile function of cardiomyocytes from H‐R. Contractility of cardiomyocytes in aerobic condition was not affected by the mixtures of tested drug combinations.

**Figure 6:**
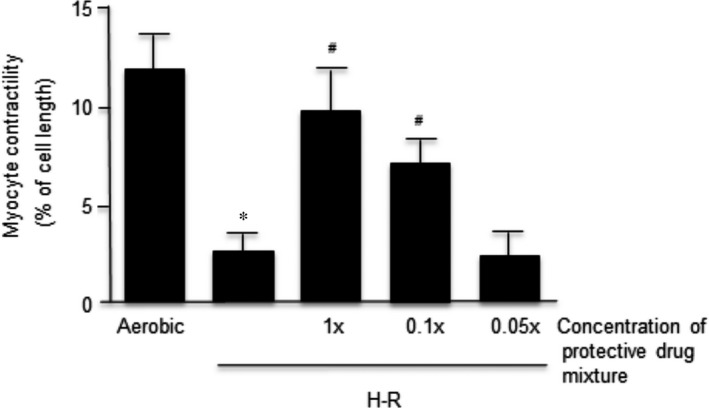
Contractility of cardiomyocytes in dose response of combined subthreshold concentrations of ML‐7, Doxy and 1400W. ‘1×’ concentration is defined as mixture of ML‐7 Doxy and 1400W with concentrations of 0.5, 10 and 25 μM, respectively. Data are expressed as mean ± S.E.M., *n* = 6/group; *P < 0.05 in comparison to aerobic control; ^#^ *P* < 0.05 in comparison to H‐R control.

Figure 6 shows that a 10‐fold dilution of the Doxy plus ML‐7 plus 1400W mixture used in Figure 5 also had a protective effect on the contractility of cardiomyocytes exposed to H‐R. However, the protective effect was lost at a 50‐fold dilution. More work remains to be done to establish to the optimal protective concentrations of these drugs.

### Effect of co‐administration of a mixture of subthreshold concentrations of inhibitors of MMP‐2, MLCK and NOS on MLC1 level in H‐R‐exposed cardiomyocytes

**Figure 7:**
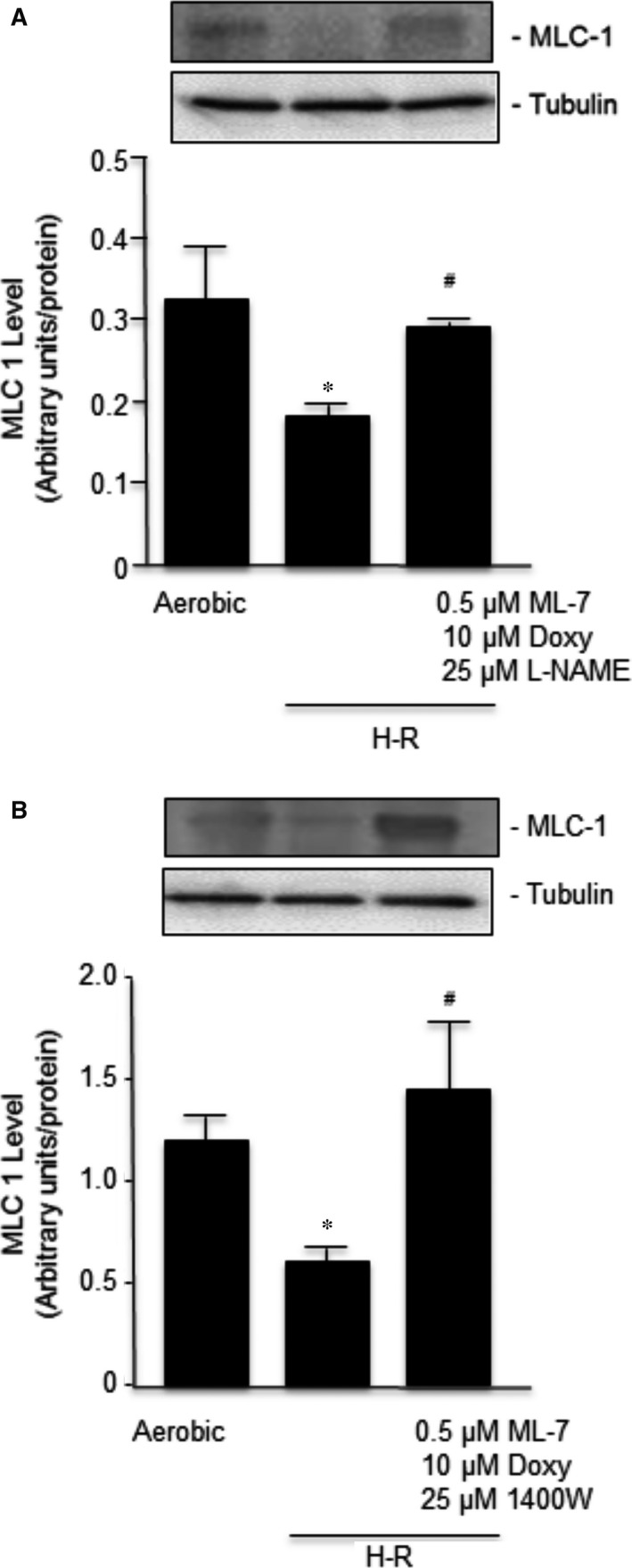
Immunoblot analysis of MLC1 level in an aerobic control group and in H‐R group subjected to drug treatment with mixtures of subthreshold concentrations of ML‐7, Doxy and L‐Name (**A**) or 1400W instead of L‐NAME (**B**). MLC1 level is expressed as densitometric units of MLC‐1 level divided by densitometric units of tubulin level. The insert shows a representative blots. Data are expressed as mean ± S.E.M., *n* = 6/group; *P < 0.05 in comparison to aerobic control; ^#^ *P* < 0.05 in comparison to H‐R control.

Myosin light chain 1 levels were measured in cardiomyocytes subjected to aerobic or H‐R conditions with or without drug treatment were analysed. Our results showed that H‐R decreases MLC1 by approximately 50% (Fig. 7) and that pretreatment of cardiomyocytes with a mixture of subthreshold concentrations of Doxy, ML‐7 and L‐NAME (Fig. 7A) or Doxy, ML‐7 and 1400W (Fig. 7B) prevented the H‐R‐induced decrease in the MLC1 levels.

## Discussion

The development of new pharmacological interventions for the prevention or treatment of I/R injury requires knowledge of the molecular fundamentals of this process. The isolated cardiac myocytes obtained from rat hearts were used to provide evidence that the intracellular causes of contractile dysfunction due to H‐R can be reduced and prevented by a pharmacological cocktail that inhibits the target molecules MMP‐2, MLCK and iNOS/eNOS.

In these studies, we have used cyanide to induce hypoxia *via* the inhibition of mitochondrial oxidative phosphorylation. Cobalt (II) chloride (CoCl_2_) is another known method to produce hypoxia *via* inhibition of prolyl hydroxylase activity with further accumulation of hypoxia‐sensitive α‐subunits of hypoxia‐inducible transcription factor‐1 (HIF‐1) 28. Liu and collaborators used 0.2 mM CoCl_2_ together with 10% or 2% O_2_ in order to induced hypoxia in mouse and human cardiomyocytes 29. They showed overexpression of iNOS *via* HIF‐1 regulation 29. Inhibition of prolyl hydrolase and induction of hypoxia‐sensitive factors can be also be achieved with iron chelators such as deferoxamine 30, but it requires a significantly longer time of hypoxia. An even different mechanism for hypoxia induction was described by Wu and collaborators 31. They used serum and glucose deprivation on the H9c2 cell line, and they showed a significant cytotoxicity, overproduction of ROS and loss of mitochondrial membrane potential 31.

We had previously shown that post‐translational modifications of contractile proteins, such as phosphorylation and nitration/nitrosylation of MLC1 and MLC2, due to an increased activation of MLCK and iNOS/eNOS, play an important role in heart contractile dysfunction after I/R 15, 16, 18. Moreover, we have documented that one of the main elements of cardiac contractile dysfunction after I/R is MMP‐2 7, 32, 33. With this knowledge, we showed that the inhibition of MLC1 phosphorylation by the MLCK inhibitor ML‐7, and the inhibition of MMP‐2 activity by Doxy allows for protection of cardiac mechanical function, improved coronary flow and recovery of cardiac contractile function after I/R 18, 25, 34. In this present study, we demonstrated a new, more effective strategy for the prevention of heart mechanical function from I/R.

It must be pointed out that MMP‐2 belongs to a family of extracellular, proteolytic enzymes which share similar substrate specificity 6, 35, 36, 37. In addition, lack of specific/selective inhibitors of MMPs makes research on MMP‐2 or other MMPs not an easy task. Nevertheless, our research and others (reviewed in: 6, 37) showed clearly that MMP‐2 plays a major role in contractile dysfunction caused by I/R, and this is why in this study we focused on MMP‐2 only. However, a contribution of other MMPs to mechanical dysfunction of the heart must be also considered.

It has been previously shown that oxidative stress plays an important role in phosphorylation and degradation of MLC1 during I/R 18, 38. Myosin light chain 1 is a main structural and functional component of muscle contractile apparatus, and thus intracellular degradation of MLC1 is associated with cardiomyopathy 7, 39. Using the isolated cardiomyocytes model from rat hearts we showed that the oxidative stress during H‐R significantly affects the contractility of isolated cardiomyocytes *in vitro*. The cardiomyocyte contractility (presented as % of cell shortening) was more than 70% lower in cells subjected to H‐R in comparison to the aerobic control. One of the mechanisms leading to decreased contractile function was an increased activity of MMP‐2. We showed that the inhibition of MMPs including MMP‐2 by protective concentrations of Doxy led to full recovery of contractile function. This confirms that MMP activity affects the contractile proteins of isolated cardiomyocytes. This remains consistent with previous studies, which showed the intracellular proteolytic role of MMP‐2 35, 40.

It was reported that MMP‐2 may act within minutes; hence, an increased proteolytic degradation of contractile proteins in an autocrine manner during 3 min. of hypoxia and 20 min. of reoxygenation seems to be likely. On the basis of previous studies showing that contractile proteins, including MLC1, are the main target for MMP‐2 during I/R 7 and that the phosphorylation of MLC1 enhances the affinity of MMP‐2 to MLC1 in isolated rat heart model 25, we subjected the isolated cardiomyocytes to different concentrations of ML‐7. The inhibition of MLC1 phosphorylation led to preserved cell contractility after H‐R due to reduction of MLC1 affinity to MMP‐2.

The first few minutes of H‐R lead to a burst of oxidative stress releasing large amount of ROS 41, 42. It is well established that highly reactive peroxynitrite (ONOO^−^), profusely produced during I/R, contributes to cardiac injury including contractile dysfunction 5, 43. As phosphorylation of MLC1 was involved in triggering MMP‐2 mediated degradation, we also examined an increased generation of nitric oxide during H‐R in isolated cardiomyocytes *in vitro*. Nitric oxide serves as a substrate for ONOO^−^ synthesis during oxidative stress; hence, it is a cause of increased nitrosylation of myocardial contractile proteins, triggering MMP‐2 mediated degradation 44. For this purpose, the nitric oxide level in cardiomyocytes subjected to H‐R has been assessed and compared to cells undergoing aerobic conditions. We showed that the oxidative stress during H‐R is a rich source of nitric oxide and nitric oxide synthesis in isolated cardiomyocytes is associated with increased induction of the high‐output iNOS or eNOS.

The use of both non‐selective (L‐NAME) and selective (1400W) inhibitors of NOS activity led to reduced synthesis of nitric oxide in cardiomyocytes subjected to H‐R. Moreover, the conditioning of cardiomyocytes with high doses (100 μM) of L‐NAME and 1400W protected the contractile proteins, leading to more the 60% recovery of contractile function after H‐R. It is also known that iNOS has nitrosylase activity and mediates cysteine S‐nitrosylation of cytoplasmic proteins 45. On the basis of the study of León *et al*. (2008) and Viappiani *et al*. (2009) showing an increased activation of MMP‐2 not *via* proteolytic activation but *via* ONOO^−^ mediated nitrosylation, and the study of Chakraborti *et al*. (2004) showing the inhibitory role of ONOO^−^ to endogenous tissue inhibitors of MMP‐2 (TIMPs), the almost full recovery of cardiomyocyte contractility in our study would be the effect of reduced nitrosylation of MLC followed by reduced MMP‐2 mediated degradation, decrease of non‐proteolytic activation of MMP‐2, decreased inhibition of TIMPs or all three together 5, 44, 46. This needs further investigation.

In the dose–response experiments, we identified the subthreshold doses for Doxy being 10 μM, for ML‐7 being 0.5 μM and for L‐NAME and 1400W being 25 μM. As shown, those concentrations, used individually, did not protect the contractile function of cardiomyocytes. However, although non‐protective doses of inhibitors did not protect the MLC1 separately, the co‐administration of subthreshold doses of three inhibitors simultaneously showed a synergistic effect leading to reduced degradation of MLC1 during H‐R and near complete recovery of cardiomyocyte contractile function. The debilitation of this effect in a dose‐dependent manner (Fig. 6) proves the synergistic effect of individually not protective concentrations of the inhibitors used. This observation proves that contractile dysfunction of myocytes after H‐R is inter alia associated with enhanced phosphorylation and nitrosylation of MLC1 and thereby an increased MMP‐2‐mediated degradation.

In summary, in this study, we demonstrated a possible novel pharmacological strategy that could be used to prevent the myocardial contractile dysfunction induced by H‐R. The simultaneous administration of inhibitors of three different pathways of H‐R‐induced cardiac contractile dysfunction fully protects cardiomyocytes from the effects of H‐R. As MMP‐2, MLCK and NOS play an important physiological role in mammalian organisms, we propose to use the combination of subthreshold, lower concentrations of inhibitors to reduce overactivation of important functional proteins. We believe that administration of low‐dose but combined pharmacological therapy might allow more effective prevention of cardiac contractile dysfunction after I/R in humans.

The lack of inhibitor selectivity/specificity is a major problem in the study of MMP biology. The other limitation is use of isolated cardiomyocyte as a model for heart injury; however, this model is ideal to address regulation of myocyte contractility by intracellular mechanisms. Despite limitations, these studies will lay the foundation for the possible development of a multi‐target ‘drug cocktail’ approach to protecting the mechanical function of the heart from oxidative stress, while lowering the risk of drug toxicity and side‐effects.

Further investigation into the consequences of contractile protein preservation following I/R in cardiomyocytes could also yield benefits in preserving the function of the heart's conduction, as well as reducing the frequency of feared complications of MI in the days to years following acute coronary syndrome. For instance, reducing the onset of infarct induced cardiac arrhythmias, ventricular free wall rupture and ventricular aneurysm could have morbidity and mortality benefits beyond that of restoring myocardial contractility alone 47, 48. Similarly, given the substantial role of I/R‐triggered injury in other bodily tissues in numerous pathological states and medical procedures, such as transplant and thrombolysis, applications of this approach could extend well beyond the heart alone.

## Conflicts of interest

The authors confirm that there are no conflicts of interest.
